# A rare case of thyroglossal duct cyst extending to the sublingual space: A case report

**DOI:** 10.1097/MD.0000000000019389

**Published:** 2020-04-24

**Authors:** Mi Jung Park, Hwa Seon Shin, Dae Seob Choi, Hye Young Choi, Ho Cheol Choi, Sang Min Lee, Jeong Ho Jang, Jeong-Hee Lee, Jung Je Park, Sung Eun Park

**Affiliations:** a Department of Radiology; b Department of Pathology; c Department of Otolaryngology, Head and Neck Surgery, Gyeongsang National University School of Medicine and Gyeongsang National University Hospital, Jinju; d Department of Radiology, Gyeongsang National University School of Medicine and Gyeongsang National University Changwon Hospital, Changwon, Republic of Korea.

**Keywords:** sublingual space, thyroglossal duct cyst

## Abstract

**Rationale:**

Thyroglossal duct cyst (TGDC) is the most common congenital anomaly of midline neck masses. A thyroglossal duct cyst is especially difficult to diagnose and is treated differently when it appears in the sublingual area. Here, we report a rare case of TGDC extending to the sublingual space.

**Patient concerns:**

A 42-year-old female presented with a history of neck swelling in the submental region.

**Diagnosis:**

The final pathologic diagnosis was a TGDC.

**Interventions:**

Sistrunk operation was performed.

**Outcomes:**

Recurrence of the disease has not been seen for the past year.

**Lession:**

Clinical awareness of the thyroglossal duct cyst in the sublingual area or on the oral floor area is important for an accurate diagnosis and the appropriated management.

## 1 Introduction

A thyroglossal duct cyst is the most common congenital anomaly, which arises from the remnants of the thyroglossal duct and occurs in 7% of the adult population.^[1]^

It commonly presents as a painless midline neck mass below the level of the hyoid bone, and it rarely occurs in the oral cavity.^[2]^ A thyroglossal duct cyst is especially difficult to diagnose when it appears in the sublingual area or on the oral floor area because it is often difficult to differentiate from other cysts such as dermoid cysts, epidermoid cysts, ranula and cystic hygromas.^[3,4]^ Preoperative imaging is important to differentiate thyroglossal duct cyst from other lesions, to confirm the diagnosis, to identify the presence of functioning thyroid tissue in the neck, and to detect any possibility of malignant change within the cyst.^[5]^

Here, we report a case of a 42-year-old female with a rare occurrence of a thyroglossal duct cyst extending to the sublingual space.

## 2 Case report

This human study was approved by Gyeongsang National University Hospital Institutional Review Board and informed consent was waived by the Institutional Review Board.

A 42-year-old female presented with a history of neck swelling in the submental region. On physical examination, a 3-cm mass was palpated in the midline submental region and was soft on palpation. The mass was mobile in the vertical direction upon swallowing. Laryngoscopy was normal.

**Figure 1:**
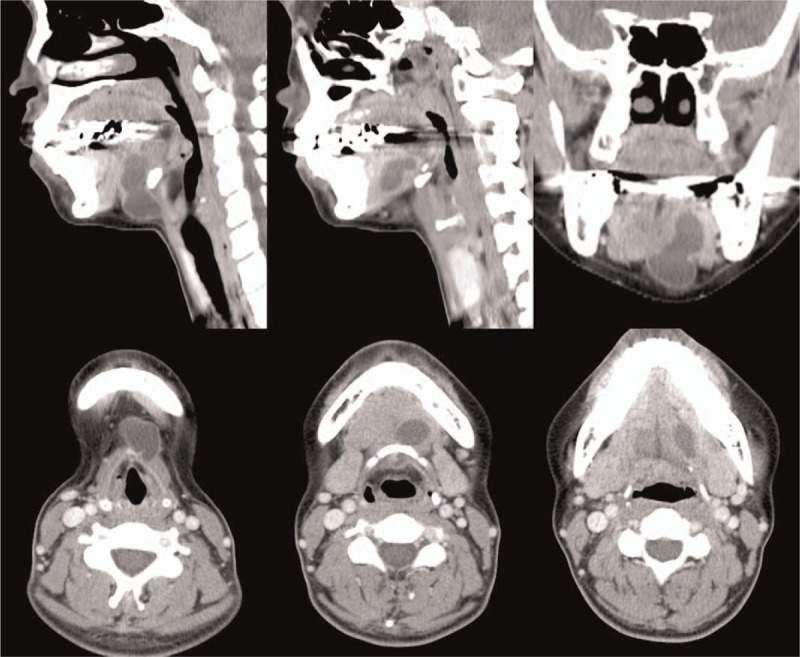
CT scan showing a well-defined thin walled cystic lesion measuring approximately 3 cm in the midline infrahyoid region extending into the hyoid bone, reaching to the left mouth floor, between the genioglossus muscle and mylohyoid muscle. The thyroid gland is within the normal position (not shown).

A CT scan showed a well-defined thin walled cystic lesion measuring 2.6×1.8×3.4 cm. The cystic lesion was located in the midline infrahyoid region extending into the hyoid bone, reaching to the left mouth floor, between the genioglossus muscle and the mylohyoid muscle (Fig. 1). Additionally, the CT scan showed that the thyroid gland was within the normal position.

**Figure 2:**
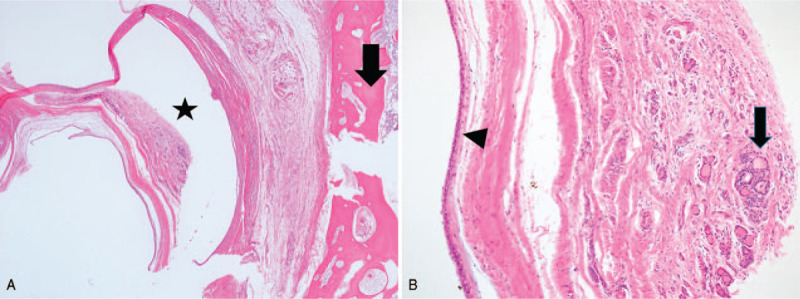
Thyroglossal duct cyst (hematoxylin eosin stain, magnification ×12.5 and ×100) A. The specimen shows the hyoid bone (arrow) and cyst (star). B. The cyst was lined with flattened squamous epithelium (arrow head) and shows thyroid tissue at the wall (arrow).

Excision of the cyst with the body of the hyoid bone and the track to the base of the tongue was performed. The specimen was sent for histopathologic examination. The histological hematoxylin and eosin sections showed flattened epithelium cells supported by a fibrocollagenous cyst wall. In a focal area of the cyst wall, there was a small portion of thyroid tissue (Fig. 2). The final pathologic diagnosis was a thyroglossal duct cyst. Postoperative recovery was uneventful, and recurrence of the disease has not been seen for the past year.

## 3 Discussion

**Figure 3:**
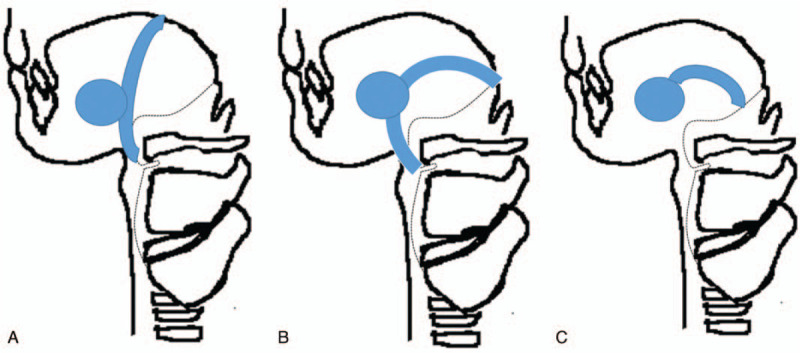
Schematic drawing of the ectopic foramen cecum (A), abnormal route of descent of the thyroid (B) and lateral branching of the thyroglossal duct (C).

**Figure 4:**
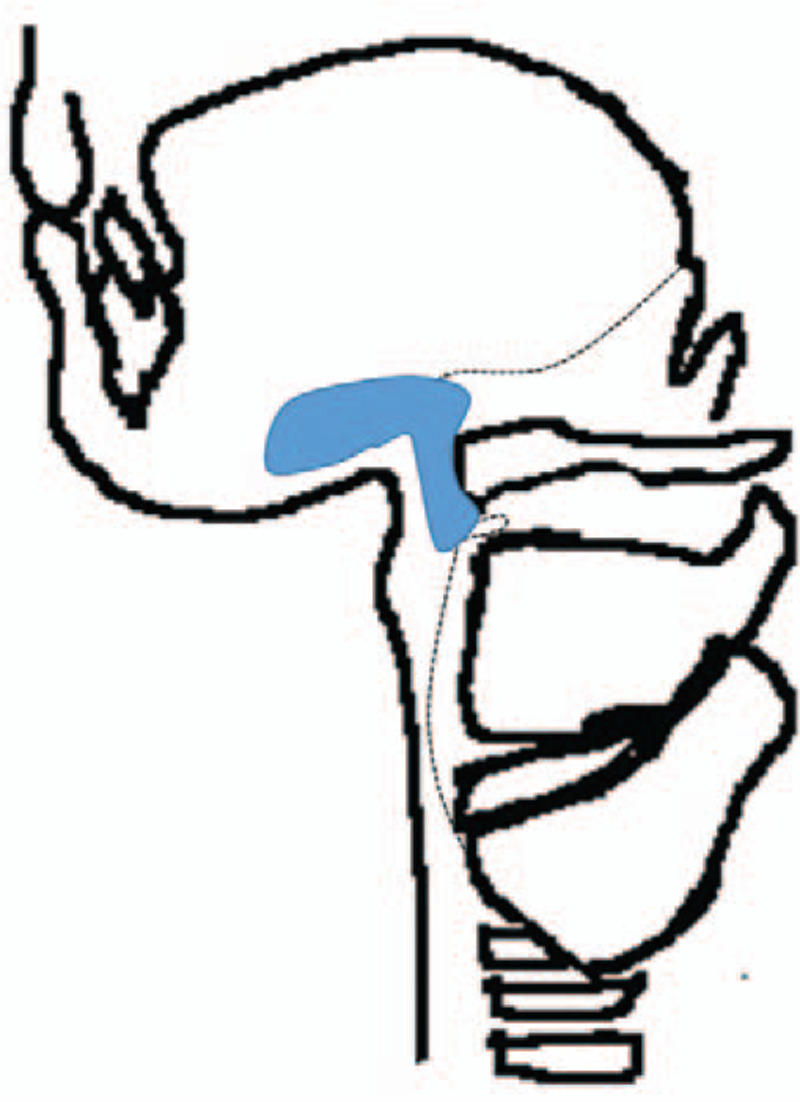
Schematic drawing of the thyroglossal duct cyst extending into the sublingual space.

Thyroglossal duct cysts can be found anywhere between the foramen cecum at the base of the tongue to the level of the suprasternal notch. There are four general locations of the thyroglossal duct cyst – intralingual (2.1%), suprahyoid (24.1%), thyrohyoid (60.9%) and suprasternal (12.9%).^[6]^ It is uncommon to find the thyroglossal duct cyst in the region of the mouth floor and sublingual gland since the floor of the mouth as well as the sublingual area is apart from the migration route of the thyroid gland. This article reports a rare case of a thyroglossal duct cyst involving the floor of the mouth and the sublingual region. Only 8 cases have been reported in the English language literature (Table 1).^[7–13]^ Associated factors observed in conjunction with occurrences in these rare sites include ectopic foramen cecum of the tongue,^[14]^ possible abnormal route of descent of the thyroid and lateral branching of the thyroglossal duct^[15]^ (Fig. 3). This case is very interesting and unique because a thyroglossal duct cyst extends into the sublingual space from the infrahyoid region, which seems to show an abnormal route of descent to the thyroid (Fig. 4).

**Table 1:**
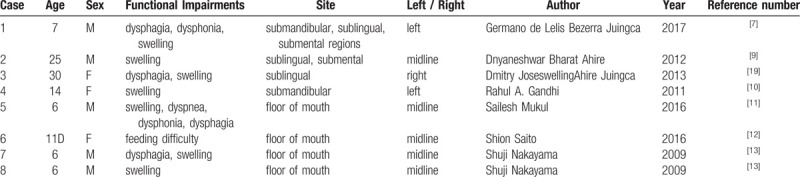
English literature reports of cases of thyroglossal duct cyst that developed on the oral floor.

A thyroglossal duct cyst classically presents as a mobile, painless mass in the midline of the neck, usually in proximity to the hyoid bone. Movement of the cyst with swallowing is often cited as a reliable diagnostic sign. Occasionally, a thyroglossal duct cyst can show atypical presentation either clinically or radiologically, which may pose a diagnostic challenge. Thyroglossal duct cysts that occur in the sublingual region or the oral floor are associated with functional impairments such as dyspnea, dysphagia, dysphonia and difficulty feeding.^[7–13]^ In our patient, there were no functional impairments, only neck swelling. In this study, sublingual extension was not suspected on clinical grounds before the CT scan, and we would have missed the sublingual lesion if we did not do a CT scan. Preoperative imaging such as CT is important to confirm the diagnosis of atypical cases, to differentiate thyroglossal duct cysts from other lesions, to identify the presence of functioning thyroid tissue in the neck and to detect any possibility of malignant change within the cyst; additionally, it plays a supplementary role in more accurately delineating the anatomy of the lesion.^[5]^ Failure to detect a thyroglossal duct cyst may be associated with inadequate performance of a surgical procedure, such as simple incisional biopsy or enucleation, both of which are associated with significant recurrence rates.^[16]^

A thyroglossal duct cyst is especially difficult to diagnose when it appears in the sublingual area or on the oral floor area because it is often difficult to differentiate from other cysts such as dermoid cysts, epidermoid cysts, ranula and cystic hygromas.^[3,4]^ Dermoid cysts are more often seen in the submandibular space than on the floor of the mouth. They are lined by keratinizing squamous epithelium and appear as well-demarcated cysts. They may contain fatty and calcific components as well as fluid. Epidermoid cysts are lined by a simple squamous epithelium and are more frequently seen in the floor of the mouth than in the submandibular space. With imaging, they appear as simple midline cystic lesions. A simple ranula is manifested clinically by swelling in the floor of the mouth and occasionally occurs in the submandibular space by either herniating through a mylohyoid defect or arising from an ectopic sublingual gland. The ruptured ranula (plugging ranula) usually extends posteriorly from the sublingual space into the submandibular space, with a narrower posteriorly from the sublingual space into. Cystic hygromas are characteristically infiltrative in nature and do not respect fascial planes typically involving contiguous anatomic regions in the neck.^[17]^

When a thyroglossal duct cyst occurs in the neck, recurrence is likely following simple enucleation; therefore, the need for concurrent resection of the hyoid bone has been suggested.^[18]^ In this case, we performed a Sistrunk operation, and there has been no recurrence to date.

## Author contributions

**Conceptualization:** Hwa Seon Shin, Jung Je Park.

**Data curation:** Mi Jung Park, Hye Young Choi, Ho Cheol Choi, Jeong-Hee Lee, Jung Je Park.

**Investigation:** Hye Young Choi, Sang Min Lee, Jeong Ho Jang.

**Methodology:** Mi Jung Park, Hwa Seon Shin, Dae Seob Choi, Jung Je Park.

**Supervision:** Dae Seob Choi.

**Validation:** Hye Young Choi.

**Writing – original draft:** Mi Jung Park.

**Writing – review & editing:** Hwa Seon Shin, Dae Seob Choi, Jeong Ho Jang, Jeong-Hee Lee, Sung Eun Park.
